# Newborn screening for haemophilia: The views of families and adults living with haemophilia in the UK

**DOI:** 10.1111/hae.13706

**Published:** 2019-02-28

**Authors:** Felicity K. Boardman, Rachel Hale, Philip J. Young

**Affiliations:** ^1^ Division of Health Sciences, Warwick Medical School University of Warwick Coventry UK; ^2^ School of Life Sciences University of Warwick Coventry UK

**Keywords:** attitudes, bloodspot, ethics, genetics, haemophilia, newborn screening, social implications

## Abstract

**Introduction:**

As genomic sequencing become more efficient and cost‐effective, the number of conditions identified through newborn screening globally is set to dramatically increase. Haemophilia is a candidate condition; however, very little is known about the attitudes of the haemophilia community towards screening.

**Aim:**

This study aimed to outline the perspectives of adults with haemophilia and their families towards newborn screening.

**Methods:**

A paper and online survey on screening were distributed to every family known to the Haemophilia Society UK. Data collection occurred between January and June 2018. In total, 327 participants completed the survey: 76% were a relative of a person with haemophilia and 24% had haemophilia themselves; 83% were living with haemophilia A and 17% with haemophilia B.

**Results:**

The vast majority supported newborn screening (77%) and preferred it to other forms of screening (preconception or prenatal). Participants supported newborn screening primarily because they viewed it as a means to facilitate early support and treatment, facilitate informed decisions about future pregnancies and prevent the “diagnostic odyssey.” The 23% who did not support the screen did not associate these particular benefits with newborn screening.

**Conclusion:**

Haemophilia emerged from this analysis as a condition that the vast majority of participants considered a “liveable” disability and one best suited to newborn screening programmes that could improve support to affected families rather than reduce the birth rate of affected children.

## 1 INTRODUCTION

Rapid developments in genomic technologies now mean that it is possible to screen newborn babies for larger numbers of conditions simultaneously than ever before. The development of tandem mass spectrometry, and the declining cost and increasing efficacy of whole genome/exome sequencing using technologies such as CRISPR‐Cas9 now mean that high output mass screens are on the horizon, prompting renewed interest in the potential population health and societal benefits of population‐based genomic screens.^1^, ^2^, ^3^, ^4^ By conducting untargeted genome/exome sequencing, whole panels of genetic conditions can now be screened for from a single blood sample, as well as child's propensity to future diseases, and even their potential reactions to certain medicines and drugs. As such, direct‐to‐consumer private genetics companies are already capitalizing on the value of this information to new parents by offering expansive newborn screens for a nominal fee.

Haemophilia is a potential candidate condition for newborn screening being expanded to the general population, who have no known family history of the condition. Affecting one in every 5000 male newborns worldwide (haemophilia A) and 1 in 30 000 male newborns (haemophilia B), it is the most severe form of X‐linked inherited bleeding disorder. Moreover, as the most serious type of bleed for those with haemophilia (intracranial haemorrhage) is most likely to occur during the neonatal period, and with about half of these babies having no family history of a bleeding disorder, newborn screening for the condition can also be justified on the grounds of protecting the health of both the infant, but also the mother who may have some bleeding symptoms.^5^

Given the perceived disease burden associated with haemophilia, however, research into population screening has previously generally focused attention on the prenatal or preconception period rather than the newborn period.^6^ However, with the advent of new treatments, particularly early prophylactic treatment that uses recombinant clotting factors, means that the disease burden of haemophilia is slowly evolving, altering the landscape of genetic screening.^7^ Indeed, the frequently observed contrast between the experiences of younger generations growing up with haemophilia and those of older generation haemophiliacs—many of whom were exposed to blood‐borne viruses such as Hepatitis B/C and HIV through contaminated blood in the 1970s and 1980s—suggests that an exploration of current attitudes towards screening for haemophilia amongst the affected community is now particularly timely.^8^, ^9^

Whilst the acceptability of newborn screens to (expectant) parents is a topic that has been widely explored in relation to a range of different conditions,^10^, ^11^, ^12^ and is a key component in the assessment of screening programmes conducted by the UK National Screening Committee, the views of affected families and adults have been less extensively researched.^10^, ^12^, ^13^, ^14^ This omission is striking given that the “hands on” direct experience possessed by these families uniquely positions them to consider what an early screen would have meant for them.^10^ Furthermore, screening has impacts for affected families that go beyond those anticipated for the general population. These include the social implications (for example, stigmatization) that come along with a shift in the “public profile” of the condition,^15^ but also a potential decrease in the condition's prevalence (as has already been observed in relation to Cystic Fibrosis since the introduction of newborn screening^16^),with associated implications for how the condition is prioritized in the context of the allocation of public funding for research into treatments.^17^

To address this identified gap in the literature, this study presents data on attitudes towards newborn genetic screening amongst people living with haemophilia A and/or B, either through having the condition themselves, or having affected relatives. By drawing on a national UK survey of families living with haemophilia, this paper contributes to an emerging area of the literature that considers the social and ethical of dimensions of screening practices from the vantage point of those already living with the disease.^10^, ^12^, ^13^, ^14^, ^16^

## 2 MATERIALS AND METHODS

This study reports on quantitative data that formed part of a larger exploratory sequential mixed methods study on attitudes to different types of screening programme across families living with various genetic conditions.^12^, ^18^, ^19^

Through the use of an advertisement in the newsletter of the UK Haemophilia Society, 22 adults with haemophilia and family members of people with haemophilia were recruited to participate in qualitative interviews that took place between April 2017 and March 2018. Seventeen interviews were conducted by telephone, and four interviews were conducted in person. The interview participants varied in terms of their ages, backgrounds and experiences with haemophilia as well co‐morbidities (associated with contaminated blood). The final sample included eight males with haemophilia and fifteen female relatives. The interviews explored participants’ experiences with haemophilia, their views on the condition's impact and their experiences of, and attitudes towards, reproductive genetic technologies. A thematic analysis was carried out on the qualitative interviews, and a survey, the Haemophilia Screening Survey (UK), directly developed from this analysis in order to measure both the significance and generalizability of the expressed ideas. The core themes were used to delineate the key domains of the survey, and, where possible, verbatim data extracts from interview participants were used to create attitude statement questions, accompanied by a likert scale. Demographic questions were replicated, or appear as modified versions of those included in the 2011 UK Census survey. Ethical approval for the survey was granted by the Biomedical and Scientific Research Ethics Committee in November 2017.

Survey data collection took place between January and June 2018. Participants were invited to complete it if they either had haemophilia A or B themselves or had the condition in their family. No restrictions were placed on the nature of the familial relationship, so step, adopted and fostered family members were all included.

A paper version of the survey was initially mailed to the 3000 households affected by haemophilia that were known to the Haemophilia Society UK, and an online version was made available and distributed through the Haemophilia Society's online networks. The link was also disseminated through the social networks associated with the research project. Participants were encouraged to distribute the survey to relevant family/friends. Postal survey returns were all processed using data scanning technology to reduce human error.

The attitudes of family members and adults with haemophilia (AwH) towards newborn genetic screening (NGS) were compared to determine whether there were any statistical differences using a chi‐squared analysis (Graphpad Prism software, v6).

## 3 RESULTS

### 3.1 Demographic data

In total, 327 people returned either an online (33/327) or postal survey (294/327). Of these, 148/327 (45%)were family members and 179/327 (55%) were AwH (Table 1); 173/327 (53%) participants were male (Table 1), including 21/148 (14%) family members and 152/179 (85%) AwH (Table 1). Overall, most participants were associated with haemophilia A (273/327, 83%), broken down into 127/148 (86%) family members and 146/179 (82%) AwH (Table 1).

**Table 1:** Characteristics and demographics of survey responders

Characteristic	All Responders (n = 327)	Families (n = 148)	AwH (n = 179)
Gender ‐ no. (%)			
Male	173 (53%)	21 (14%)	152 (85%)
Female	154 (47%)	127 (86%)	27 (15%)
Age			
18‐25 y	11 (3%)	1 (1%)	10 (6%)
26‐34 y	38 (12%)	22 (15%)	16 (9%)
35‐45 y	68 (21%)	45 (30%)	23 (13%)
46‐55 y	60 (18%)	21 (14%)	39 (22%)
56‐65 y	65 (20%)	30 (20%)	35 (19%)
>65 y	85 (26%)	29 (20%)	56 (31%)
Qualifications			
Degree or higher	142 (43%)	70 (47%)	72 (40%)
Other/none	185 (57%)	78 (53%)	107 (60%)
Religious			
Yes	183 (56%)	88 (60%)	95 (53%)
No	130 (40%)	55 (37%)	75 (42%)
Prefer not to say	14 (4%)	5 (3%)	9 (5%)
Parents			
Yes	253 (77%)	135 (91%)	118 (66%)
No	73 (22%)	12 (8%)	61 (24%)
Prefer not to say	1 (1%)	1 (1%)	0
Type of Haemophilia			
Haemophilia A	273 (83%)	127 (86%)	146 (82%)
Haemophilia B	54 (17%)	21 (14%)	33 (18%)

Demographics are shown for all responders (n = 327), responders associated with haemophilia families (families; n = 148) and adults with haemophilia (AwH; n = 179)

Variables with significant differences (*P* < 0.05) are highlighted.

### 3.2 Newborn genetic screening (NGS)

Overall, 253/327 (77%) supported NGS. However, there was a tendency towards less support for NGS in AwH: 132/179 (74%) than family members (Families: 121/148 (82%); Table 2).

**Table 2:** Response summaries for questions assessing views on newborn genetic screening (NGS)

Question	All Responders (n = 327)	Families (n = 148)	AwH (n = 179)	F v AwH (chi‐2)
				P Value
Identifying haemophilia/bleeding disorders at birth would lead to better support and health care for the child and their family				
Agree	291 (89%)	134 (91%)	157 (88%)	0.66
Other	36 (11%)	14 (9%)	22 (12%)	
Identifying haemophilia/bleeding disorders at birth would extend the life expectancy of a child with a bleeding disorder				
Agree	212 (65%)	91 (61%)	121 (68%)	0.24
Other	115 (35%)	57 (39%)	58 (32%)	
Identifying haemophilia/bleeding disorders at birth (and not in pregnancy) takes away the parents right to make a decision about whether or not they want to have a child with a bleeding disorder				
Agree	107 (33%)	44 (30%)	63 (35%)	0.29
Other	220 (67%)	104 (70%)	116 (65%)	
Identifying haemophilia/bleeding disorders before a child develops any symptoms prevents the child and their family from enjoying life whilst they are still symptom‐free				
Agree	96 (29%)	38 (26%)	58 (32%)	0.18
Other	231 (71%)	110 (74%)	121 (68%)	
Identifying haemophilia/bleeding disorders at birth would help research into a cure by enabling more children to be enrolled into clinical trials early on				
Agree	198 (61%)	90 (61%)	108 (60%)	0.93
Other	129 (39%)	58 (39%)	71 (40%)	
Identifying haemophilia/bleeding disorders at birth would interfere with the early bonding process between parent and child				
Agree	18 (6%)	10 (7%)	8 (4%)	0.36
Other	309 (94%)	138 (93%)	171 (96%)	
Identifying haemophilia/bleeding disorders at birth would make the diagnosis easier for parents to accept				
Agree	177 (54%)	74 (50%)	103 (58%)	0.17
Other	150 (46%)	74 (50%)	76 (42%)	
Identifying haemophilia/bleeding disorders at birth would spare parents the difficulties associated with finding a diagnosis for the child later on				
Agree	254 (78%)	118 (80%)	136 (76%)	0.41
Other	73 (22%)	30 (20%)	43 (24%)	
Even if parents could not know for sure the severety of the haemophilia/bleeding disorder affecting their newborn baby, its still better that they know about the bleeding disorder straight away				
Agree	284 (87%)	130 (88%)	154 (86%)	0.63
Other	43 (13%)	18 (12%)	25 (14%)	
Identifying haemophilia/bleeding disorders at birth is important as it would enable parents to make informed decisions about future pregnancies				
Agree	266 (81%)	123 (83%)	143 (80%)	0.45
Other	61 (19%)	25 (17%)	36 (20%)	
It is unethical not to screen newborn babies for conditions that can be treated				
Agree	144 (44%)	67 (45%)	77 (43%)	0.68
Other	183 (56%)	81 (55%)	102 (57%)	
I would support a newborn genetic screening programme for haemophilia/bleeding disorders				
Agree	253 (77%)	121 (82%)	132 (74%)	0.08
Other	74 (23%)	27 (18%)	47 (26%)	

Response breakdowns are shown for families and AwH. Responses for each question were stratified as “agree” v “other” (other=disagree and neither disagree nor agree).

The key reasons driving support for NGS was a belief that it would lead to better support, would allow parents to make informed decisions about future pregnancies and would spare parents the difficulties associated with a later diagnosis (Table 2). Participants also believed than an early screen could extend life expectancy and would allow earlier enrolment on clinical trials (Table 2). Most participants agreed that NGS is important even if severity could not be determined accurately at birth in all cases (Table 2).

Less than half of the participants agreed that NGS is unethical because of the lack of curative treatment available (a common ethical argument used against the introduction of NGS) or the notion that by using NGS, and not other forms of screening (such as preconception or prenatal screening), that NGS denies parents the choice as to whether or not they want to bring children affected by haemophilia into the world in the first place (Table 2). Similarly, less than half the sample agreed that newborn screening would stop families and children enjoying life whilst they were still symptom‐free and most disagreed with the idea that receipt of a serious diagnosis early in life would interfere with parent‐child bonding (Table 2).

Interestingly, no significant differences in support for NGS were observed between those participants who came from families with multiple (>2) affected members as compared to those families who had only one or two members with haemophilia (Table 3).

**Table 3:** Support for NGS among families associated with increased numbers of people with haemophilia

	Other	Agree	Total
Associated with <2 affected individuals	42 (22%)	149 (78%)	191
Associated with 2 + affected individuals	32 (24%)	104 (76%)	136
*P* = 0.74			327

The support was compared for responders associated with <2 affected individuals compared with those associated with 2 + affected individuals. Numbers and overall percentages are shown.

Differences were assessed using chi‐^2^ analysis (*P*‐value).

74/327 (23%) responders did not support NGS: 27/148 (18%) families and 47/179 (26%) AwH (Table 4). Sub‐analysis of these responders highlights that in general they did not believe introducing NGS would extend life expectancy or increase enrolment on clinical trial. However, although this 23% of responders did not personally support NGS introduction, they did not believe NGS would reduce presymptomatic quality of life, interfere with the parent‐child bonding process, make the diagnosis easier to accept for the parents or believe that it is unethical to screen newborn babies for diseases that cannot be treated (Table 4).

**Table 4:** Response summaries for responders who did not support the introduction of NGS. Response breakdowns are shown for families and AwH

Question	Responders who do not support NGS			
	All Responders (n = 74)	Families (n = 27)	AwH (n = 47)	F v AwH
				*P* Value
Identifying haemophilia/bleeding disorders at birth would lead to better support and health care for the child and their family				
Agree	44 (59%)	15 (56%)	29 (62%)	0.63
Other	30 (41%)	12 (44%)	18 (38%)	
Identifying haemophilia/bleeding disorders at birth would extend the life expectancy of a child with a bleeding disorder				
Agree	34 (46%)	13 (48%)	21 (45%)	0.81
Other	40 (54%)	14 (52%)	26 (55%)	
Identifying haemophilia/bleeding disorders at birth (and not in pregnancy) takes away the parents right to make a decision about whether or not they want to have a child with a bleeding disorder				
Agree	17 (23%)	5 (19%)	12 (26%)	0.57
Other	57 (77%)	22 (81%)	35 (74%)	
Identifying haemophilia/bleeding disorders before a child develops any symptoms prevents the child and their family from enjoying life whilst they are still symptom‐free				
Agree	15 (20%)	5 (19%)	10 (21%)	0.99
Other	59 (80%)	22 (81%)	37 (79%)	
Identifying haemophilia/bleeding disorders at birth would help research into a cure by enabling more children to be enrolled into clinical trials early on				
Agree	18 (24%)	5 (19%)	13 (28%)	0.41
Other	56 (76%)	22 (81%)	34 (72%)	
Identifying haemophilia/bleeding disorders at birth would interfere with the early bonding process between parent and child				
Agree	6 (8%)	2 (7%)	4 (9%)	0.99
Other	68 (92%)	25 (93%)	43 (91%)	
Identifying haemophilia/bleeding disorders at birth would make the diagnosis easier for parents to accept				
Agree	22 (30%)	7 (26%)	15 (32%)	0.79
Other	52 (70%)	20 (74%)	32 (68%)	
Identifying haemophilia/bleeding disorders at birth would spare parents the difficulties associated with finding a diagnosis for the child later on				
Agree	30 (41%)	11 (41%)	19 (40%)	0.99
Other	44 (59%)	16 (59%)	28 (60%)	
Even if parents could not know for sure the severety of the haemophilia/bleeding disorder affecting their newborn baby, its still better that they know about the bleeding disorder straight away				
Agree	40 (54%)	13 (48%)	27 (57%)	0.47
Other	34 (46%)	14 (52%)	20 (43%)	
Identifying haemophilia/bleeding disorders at birth is important as it would enable parents to make informed decisions about future pregnancies				
Agree	39 (53%)	16 (59%)	23 (49%)	0.47
Other	35 (47%)	11 (41%)	24 (51%)	
It is unethical not to screen newborn babies for conditions that can be treated				
Agree	10 (14%)	4 (15%)	6 (13%)	0.99
Other	64 (86%)	23 (85%)	41 (87%)	

Responses for each question were stratified as “agree” v “other” (other = disagree and neither disagree nor agree).

Differences were assessed using chi‐^2^ analysis (*P*‐value).

## 4 DISCUSSION

This study, to the best of our knowledge, is the first to explore attitudes towards newborn screening amongst families and AwH. It has revealed that most adults with, and families affected by, haemophilia are in favour of newborn screening for the condition. With the increasing use of whole genome sequencing techniques for screening purposes, the introduction of newborn screening for conditions such as haemophilia could mark the advent of a new era in the management of the condition, including the possibility of using new non‐factor drugs on these patients with very early diagnoses. Indeed, even the 23% of participants who did not support newborn screening in this study did so not because they necessarily held negative beliefs about newborn screening (for example, that it may affect parent/child bonding or extend the illness into the presymptomatic period) but rather because they were unconvinced that newborn screening would confer the particular advantages cited by screening supporters. As such, this study did not uncover overtly negative views about this type of screening programme.

There were some differences, however, in responses between AwH, who are mostly male, and responding family members, who are mostly female, with female relatives more likely to support NGS than affected males; although these differences are not significant, they are approaching significance (*P* = 0.08). It is possible that this difference highlights the scepticism on the part of AwH regarding any tangible differences that NGS would have made to their own lives, particularly as none of them had benefitted from the early interventions offered to boys with haemophilia today, such as the commencement of prophylaxis. Indeed, many had, in fact, developed other serious co‐morbidities during the course of their treatment, such as Hepatitis B/C or HIV, which may have also influenced their perceptions of medical interventions.

The female relatives of AwH, however, viewed NGS from an entirely different vantage point. While not having experienced haemophilia directly themselves, the female relatives were nevertheless more heavily implicated, both socially as mothers, but also physically, as carriers, in their reproductive outcomes than their male counterparts, which may have affected their perceptions of their responsibilities with regards to their family's health.^20^ It is also possible that such family members may also have a more clear memory of a diagnostic odyssey, associated with later diagnosis, and may thus more clearly be able to envisage the benefits of early identification for boys with haemophilia. We found similar differences between family members and AwH in our work on attitudes to preconception and prenatal screening for haemophilia.^19^

In spite of these differences between AwH and family members, however, support for NGS was nevertheless high. These findings, when interpreted alongside the qualitative data, suggest that the participants do not view haemophilia (even in its most severe form) to be a condition that justifies selective reproduction through preconception or prenatal screening programmes. Rather, our data suggest that participants perceived haemophilia as a “liveable” condition, and emphasized the importance of early identification (even if severity is initially unknown) in order to minimize the time to diagnosis and treatment, as well as to reduce the risks of intracranial bleeds and joint damage that can be associated with untreated haemophilia.^6^

These findings are in line with our previous work assessing support for prenatal screening for haemophilia, which found that affected adults and their family members in support of this form of screening do so not because they believe that selective pregnancy termination is an essential option, but rather because they believe that prenatal screening can provide vital information to prepare for the birth of a haemophiliac child, and also to protect the carrier mother.^5^

However, in spite of this scepticism around the reproductive value of screening for haemophilia, there was nevertheless support for the idea that NGS could be used by the parents of already affected children, to inform decisions about their future pregnancies. This finding is noteworthy as it suggests that the reproductive decisions and attitudes of already affected families are viewed differently by the haemophilia community than those made by the general population, highlighting the positive way that “experiential knowledge” and insight are valued in the appraisal of future affected lives.^21^, ^22^ Indeed, the families and adults who participated in this survey had direct—and often extensive—knowledge of life with haemophilia, with 136/327 participants (42%) (Table 3) of the sample having more than two people in their family living with haemophilia—a much higher rate of inter‐family recurrence than has been noted by studies of screening attitudes within families affected by other genetic conditions.^21^

Overall, therefore, this study highlights the rich and complex insights that families living with genetic disorders bring to debates around expansive screening programmes. It is critical that any screening programme for haemophilia has an infrastructure that is able to capture and accurately reflect the reality of life with the condition, as this is likely to be different to the perceptions of haemophilia within the general public.^22^ The inclusion of families and AwH into screening policy debates is a key mechanism by which this change can occur.

### 4.1 Further research/Policy implications

This study underscores the importance of consulting affected families when evaluating and implementing genetic screening programmes. Indeed, these groups have much to offer an understanding of the realities of genetic impairment, a form of knowledge that will only become more significant as genomic medicine expands and decisions will need to be made regarding which conditions should be included on genetic screening panels, and which should not. Indeed, further research could usefully explore the knowledge and views of the general public towards screening for haemophilia in order to identify differences and similarities in their perceptions of the condition, as well as to inform and facilitate the development of mechanisms of decision support as reproductive decisions become invariably more complex for the whole of society.

### 4.2 Strengths and weaknesses

Recruiting through a support group, and not clinics, may have imposed sample bias to the analysis as support groups are more likely to attract people experiencing difficulty, and those who value particular forms of support. Indeed, it is noteworthy that a high proportion of women with haemophilia (15%) participated in the study, which might be explained by the methods of recruitment, given that support groups are disproportionately accessed by women. Moreover, due to confidentiality and data protection requirements, no identifiable information was asked of participants. Whilst this may have aided recruitment, there was no means by which to prevent a participant from completing multiple surveys. In spite of these weaknesses, however, the final sample was nevertheless diverse.

Given that participants were being asked about a hypothetical (rather than already implemented) screening programme, it is also possible that participants’ prior knowledge of NGS was limited, a fact that may have skewed their attitudes towards it. However, the current high profile of genomic medicine within the public and policy arena, the fact that all participants had prior experience of a genetic disease in their family as well as the relatively recent introduction (2006) of an NGS in the UK for another genetic condition, Cystic Fibrosis, together meant that participants did not have to make large imaginative leaps to envisage the potential transferability of NGS to haemophilia, and as such, we do not feel that this lack of background knowledge need limit the value of the data.

## DISCLOSURE

The authors stated that they had no interests which might be perceived as posing a conflict or bias.
